# Numerical Modeling of the Electronic and Electrical Characteristics of InGaN/GaN-MQW Solar Cells

**DOI:** 10.3390/ma12081241

**Published:** 2019-04-16

**Authors:** Bilel Chouchen, Mohamed Hichem Gazzah, Abdullah Bajahzar, Hafedh Belmabrouk

**Affiliations:** 1 Quantum and Statistical Physics Laboratory, Faculty of Sciences of Monastir, University of Monastir, Monastir 5019, Tunisia; bilelchouchen06@gmail.com (B.C.); Hichem.Gazzah@fsm.rnu.tn (M.H.G.); 2 Department of Computer Science and Information, College of Science, Majmaah University, Zulfi 11932, Saudi Arabia; a.bajahzar@mu.edu.sa; 3 Electronics and Microelectronics Laboratory, Faculty of Science of Monastir, University of Monastir, Monastir 5019, Tunisia; 4 Department of Physics, College of Science, Majmaah University, Al Zulfi 11932, Saudi Arabia

**Keywords:** InGaN/GaN-MQW, solar cells, temperature, polarization, conversion efficiency

## Abstract

In this paper, a numerical model allows to analyze the photovoltaic parameters according to the electronic properties of In_x_Ga_1−x_N/GaN MQW solar cells under the effect of temperature, the number of quantum wells and indium composition. The numerical investigation starts from the evaluation through the finite difference (FDM) simulation of the self-consistent method coupled with the photovoltaic parameters taking into account the effects of the spontaneous and piezoelectric polarization. The results found were consistent with the literature. As expected, the temperature had a negative impact on the performance of InGaN/GaN MQW solar cells. However, increasing the number of quantum wells improves cell performance. This positive impact further improves with the increase in the indium rate. The obtained results were 28 mA/cm^2^ for the short-circuit current density, 1.43 V for the open-circuit voltage, and the obtained conversion efficiency was 31% for a model structure based on 50-period InGaN/GaN-MQW-SC under 1-sun AM1.5G.

## 1. Introduction

The excellent material properties make III-V semiconductors attractive for applications in optoelectronics [1] and advantageous for the manufacture of solar cells at high performance. InGaN alloys have many promising characteristics for photovoltaic applications, such as tunable direct band gap ranging from the visible to ultraviolet spectrum, and the large absorption coefficient [2]. In addition, InGaN alloys have the advantages of high carrier mobility, high absorption coefficient and excellent thermal/radiation resistance. These characteristics contribute to the achievement of highly efficient solar cells for potential use at elevated temperatures [3,4]. The insertion of quantum wells into the intrinsic region of a p-i-n solar cell improves the energy efficiency of the solar cell [5,6]. Thanks to the high efficiency of their solar cells, InGaN/GaN multiple quantum well (MQW) heterostructures have been extensively studied, through the variation of different parameters.

Temperature is one of the most important factors when using solar cells. In fact, when the light intensity rises, it will cause an increase in the heat generated by the poor conversion of the photons into electricity in the intrinsic region, which is accompanied by a reduction in the efficiency of the cell. It is, thus, important to understand the thermal stress properties of InGaN QW solar cells [7]. Few in-depth studies underlined the interest of the thermal properties of the InGaN-MQW solar cell [8]. Their results show that the increase in temperature decreases the efficiency η and the open circuit potential Voc. On the contrary, the short circuit current density Jsc increases. The dependence of solar cells parameters on temperature with different structure types, such as p-MQW-n and p-i-n solar cells, were investigated theoretically by Asghari et al. [9] A maximum quantum efficiency around 35% was obtained under 1-sun AM1.5G condition at room temperature. They concluded, that the efficiency of MQW solar cell is much higher than that of the reference p-i-n cells at high temperature. Jeng et al. [10] studied the impact of the performance InGaN-MQW solar cells on temperature, ranging from 25 °C to 340 °C.

Several parameters can improve the photovoltaic conversion of InGaN-MQW solar cells. With regard to the type of active region, numerous groups have dedicated their research to optimizing the photovoltaic characteristics of the InGaN/GaN MQW solar cell (SC) by varying the thickness of the quantum well [11], the thickness of the barrier [12,13], the indium content of the wells [14], and the number of QWs [15,16]. Wierer et al. [13] studied the structure of InGaN/GaN MQW SC containing 15 QWs, 2.7 nm-thick In_0.21_Ga_0.79_N wells and three barriers of different GaN thicknesses of 3.0 nm, 6.3 nm and 10.0 nm. Mukhtarova et al. [17] investigated the effect of the active region thickness of In_0.1_Ga _0.9_N/GaN MQW SC. They have noted that the increase of the number of quantum wells from 5 to 40 improves the short-circuit current and the open-circuit voltage. Consequently, the photovoltaic efficiency has increased from 0.09% to 0.85%. However, beyond this number of wells, the efficiency of the device begins to drops. Deng et al. [18] have shown that increasing the quantum well thickness and the number of wells can improve cell efficiency. The optimal indium composition of the quantum well was 0.6. In this context, Belghouthi et al. [19] also reported that the maximum efficiency reaches 19% for 30-MQW In_0.6_Ga_0.4_N/GaN SC of N-face configuration. They have concluded that the fact of taking into consideration the polarization effect is a good choice to improve cell performance. 

A better understanding of the electronic, optical and thermal properties of InGaN/GaN heterostructures is necessary and has a significant impact on the physical and electrical performance of electronic components, circuits, and systems [20,21,22]. Generally, theoretical modeling and numerical simulation studies have been widely used in the photovoltaic field in order to determine the most important parameters for solar cell operation and to minimize losses and to optimize the physical and geometrical parameters in order to get maximum efficiency. In fact, these studies were based on two different approaches: On the one hand, several studies have examined the electrical parameters of the solar cell without taking into account the impact of the electronic properties of the materials [9,10,18,19]. On the other hand, other works [23,24] have been established on analytical models to take into account some electronic properties. Despite this, their analytical models have weaknesses in such a way that they have not coupled all the electronic properties with the parameters of the solar cell.

The objective of this work is to investigate the impact of the temperature, indium fraction and quantum well number effects on the electronic properties and electrical parameters in InGaN/GaN MQWs solar cells with N-face polarity. For this purpose, the numerical modeling developed was carried out by solving the one-dimensional self-consistent Schrödinger–Poisson equations considering the thermal effects and including the polarization induced charges. The consequences of this model are highlighted by the temperature dependency of effective mass, dielectric constant and the conduction band offset. This paper starts with a short Introduction. Then, Section 2 presents the investigated structure and the basic equations. In Section 3, we discuss the results obtained in the context of this numerical model against that reported in the literature. The final Section 4 is a conclusion.

## 2. Governing Equations and Numerical Method

### 2.1. Description of the Structure

**Figure 1:**
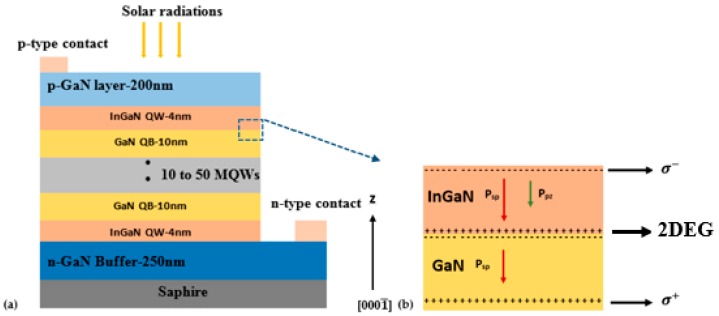
(**a**) Schematic section of the device structure model. (**b**) the cross section of the spontaneous and piezoelectric polarization charge distribution of the InGaN/GaN structure in N-face polarity.

Our reference structure is a p-GaN-In_x_Ga_1−x_N (MQW)-nGaN solar cell. The device structure is shown in Figure 1. This solar cell is characterized by a n-GaN type layer with a thickness of 250 nm and a p-GaN layer with a thickness of 200 nm.

In order to validate the results, we compared this structure against that of Deng et al. [18]. We chose the number of quantum wells ranging from 10 to 50-periods of InGaN-QW and GaN-QB barriers. The thickness of each QW is 4 nm and the thickness of the GaN-QB barrier is 10 nm. The doping concentrations in the p-type and n-type regions are, respectively, N_A_ = 2 × 10^17^ cm^−3^ and N_D_ = 2 × 10^18^ cm^−3^. The cell is illuminated by a polychromatic light under AM1.5 conditions. It should be mentioned that no external potential is applied to solar cells. The spontaneous and piezoelectric polarization vectors in the interface InGaN/GaN heterostructure in polarity N-Face are shown in Figure 1b. The InGaN layer is therefore under tensile strain. This is due to fact that the lattice parameters of the InGaN layer are lower than those of the GaN layer. In consequence, a density of positive charges and a strain induced polarization electric field at the InGaN/GaN interface is obtained. This positive charge is compensated by the presence of electrons at the interface forming a gas confined to this interface which is called two-dimensional electron gas (or 2DEG) [19]. 

### 2.2. Electrical Parameters of the InGaN/GaN MQW-SC

The relationship between current density J and voltage V for a MQW solar cell is presented by [25]:

(1) $$J\left(V\right)\;=\;J_{QW0}\left[1+r_{R}\beta \right]\left[exp\left(\frac{qV}{k_{B}T}\right)-1\right]-J_{ph}$$

where $J_{QW0}$ is the saturation current density, $J_{ph}$ is the photocurrent density, $k_{B}$ is the Boltzmann constant, q is the elementary charge, T is the temperature, $r_{R}$ is the radiative enhancement rate and β is the ratio of the current required to feed radiative recombination in the intrinsic region at equilibrium to the usual reverse drift current resulting from minority carrier extraction [25]. The term $r_{R}\beta$ accounts for the radiative recombination within the intrinsic region which has been developed by Anderson [25]. Only radiative recombination is considered in this study [19].

The definition of the quantities involved in the above equation will be presented below. The saturation current density $J_{QW0}$ is given by [24]:

(2) $$J_{QW0}\;=\;\frac{qD_{n}}{L_{n}}n_{p0}+\frac{qD_{p}}{L_{p}}p_{n0}$$

$n_{p0}\;$ and $\;p_{n0}$ are the thermal equilibrium electron and holes concentration in n-type and p-type semiconductor respectively. 

We have $n_{p0}\gg p_{n0}$ which means that the thermal equilibrium electron concentration in the p-type semiconductor dominates the current characteristics. The second term in Equation (2) is neglected. 

The temperature dependence of the carrier diffusion coefficients of electron can be calculated by:

$$D_{n}\left(T\right)\;=\;\frac{kT\mu_{e}\left(T\right)}{e}$$

$\tau_{n}$ is the lifetime of electron. In this simulation the lifetime of the carriers for In_x_Ga_1−x_N is equal to $\tau_{n}\;=\;6.5\;ns$ [10]. $L_{n}$ is the diffusion length for electron. It is expressed by: $L_{n}\;=\;{\left(D_{n}\times \tau_{n}\right)}^{0.5}$. In our simulation, the value of $L_{n}$ for In_0.2_Ga_0.8_N decreases from 5.4 μm to 4.22 μm with increasing temperature from 300 to 600 K.

$\mu_{e}\left(T\right)$ is the electron mobility. Its expression is available in [26].

The thermal equilibrium electron concentration in n-type semiconductor reads:

(3) $$n_{p0}\;=\;n_{i}exp\left(\frac{E_{i}-E_{F}}{k_{B}T}\right)$$

$n_{i}$ is the intrinsic carrier density, $E_{i}$ is the intrinsic energy, $E_{F}$ is the Fermi energy. The approach adopted to compute this quantity will be explained hereafter. 

The radiative enhancement rate $r_{R}$ represents the fractional increase in recombination in the i-MQW region, and it can be expressed by Anderson [25]:

(4) $$r_{R}\;=\;1+f_{W}\left[\gamma_{B}\gamma_{DoS}^{2}exp\left(\frac{E_{g}^{GaN}\left(T\right)-E_{g}^{InGaN}\left(T,x\right)}{k_{B}T}\right)-1\right]$$

where $f_{W}\;=\;0.5$ denotes the occupation in the i-InGaN MQW region, $\gamma_{B}\;=\;10$ the oscillator enhancement factor and $\gamma_{DoS}$ the density-of-states enhancement factor. 

The ratio of current β is related to the i-MQW active region. It is given by:

$$\beta \;=\;\frac{qW\;B_{B}\;n_{i}^{2}}{J_{QW0}}$$

with $W\;=\;N_{W}\left(L_{W}+L_{b}\right)$, W is the width of the i-MQW region. $L_{b}$ is the width of a quantum barrier, $L_{W}$ is the width of a QW and $N_{W}$ is the number of QWs. $B_{B}\;=\;10^{-10}{\;\mathrm{cm}}^{3}s^{-1}$ represents the recombination coefficient and $n_{i}$ is the intrinsic carrier density in the i-MQW region.

(5) $$n_{i}\;=\;g_{B}exp\left(\frac{E_{B}}{nk_{B}T}\right)$$

where $g_{B}\;=\;\frac{2}{h^{3}}{\left(\frac{m_{h}m_{e}}{m_{0}}\right)}^{3/4}{\left(2\pi m_{0}k_{B}T\right)}^{3/2}$ and n is the ideality factor. It is equal to 2 [10]. $E_{B}$ represents the barrier height at the InGaN/GaN interface, $m_{0}$ is the free mass of the electron, $m_{h\;}$ is the electron effective mass and $m_{e}$ is the hole effective mass [27].

The photocurrent density generated by incident photons is expressed by:

(6) $$J_{ph}\;=\;q\phi_{A}\left(T,x\right)$$

According to Deng et al. [18], the flux of photons absorbed by the MQW-region is given by:

(7) $$\phi_{A}\left(T,x\right)\;=\;N_{W}\underset{n}{\sum }N_{ph}(\lambda_{n})exp\left[\alpha_{W}(\lambda_{n},T,x\right)L_{W}]\Delta \lambda_{n}$$

$N_{ph}(\lambda_{n})$ is a quantity associated to the solar spectrum, $\lambda_{n}$ is the photon wavelength and $\Delta \lambda_{n}$ is the linewidth. Deng et al. [18] suggest calculating the absorption coefficient $\alpha_{W}(\lambda_{n})$ as follows:

(8) $$\alpha_{W}\left(\lambda_{n},T,x\right)\;=\;2.2\times 10^{5}\sqrt{\frac{hc}{\lambda_{n}}-E_{g}^{InGaN}\left(T,x\right)}$$

where h is the Planck constant.

The open circuit voltage is obtained by setting J = 0. Its expression reads:

(9) $$V_{oc}\;=\;\frac{k_{B}T}{q}\left\{Ln\left(\frac{J_{ph}+J_{QW0}\left(1+r_{R}\beta \right)}{J_{QW0}\left(1+r_{R}\beta \right)}\right)\right\}$$

The photovoltaic cell efficiency η is expressed as follows:

(10) $$\eta \;=\;\frac{P_{max}}{P_{in}}\;=\;\frac{V_{oc}FFJ_{ph}}{P_{in}}$$

where $P_{max}$ is maximal output power, $P_{in}\;=\;1000\;W.m^{-2}$ is the incident power under AM1.5G conditions and FF is the fill factor. To compute the electrical parameters of the InGaN/GaN MQW-SC, it is necessary to provide the Fermi energy $E_{F}$, the temperature T and the indium content x. 

The next paragraph will explain how to calculate the Fermi energy using a self-consistent model based on the coupled Schrodinger and Poisson equations.

In the matter of the temperature, many approaches are traditionally adopted to investigate the thermal effects on optoelectronic and microelectronic devices. The first approach consists to regard the temperature as an external parameter. The evolution of the physical parameters such as the band gap energy or the dielectric permittivity versus the temperature is expressed using empirical relations. This way of seeing the effect of the temperature has the advantage of being simple to handle. However, it assumes implicitly that the device operates at isothermal conditions. This may requires a cooling or air-conditioning system.

The second approach considers the temperature as a quantity that is affected by the device operating characteristics and inversely it has an influence on the behavior of the device. This two-way relationship requires to solve the heat equation simultaneously with the other balance equations. The coupling and mutual effects are performed via the source term due to the Joule effect in the heat equation and conversely via the variation of the physical quantities upon the temperature. The second approach has been adopted by the authors to investigate heat transfer in MOSFET [28,29,30]. It has also been used to investigate an InGaN heterostructures [31].

In the present paper, the first approach will be followed and the investigated solar cell will be assumed to operate at a constant temperature T.

### 2.3. Poisson-Schrodinger Equations:

In the framework of effective mass theory, the subband structure of the InGaN/GaN heterostructure is investigated by solving simultaneously the Schrödinger and Poisson equations [31]:

(11) $$-\frac{\hbar^{2}}{2}\frac{d\;}{dz}\left(\frac{1}{m^{*}\left(z\right)}\frac{d\psi_{\nu ,k_{z}}\left(z\right)}{dz}\right)+E_{c}\left(z\right)\psi_{\nu ,k_{z}}\left(z\right)\;=\;E_{v}\psi_{\nu ,k_{z}}\left(z\right)$$

(12) $$\varepsilon_{0}\frac{d}{dz}\left(\varepsilon_{r}\left(T,x\right)\frac{d\left(V_{H}+V_{P}\left(x\right)\right)}{dz}\right)\;=\;e^{2}\left(\frac{\sigma_{s}}{e}\delta \left(z-z_{0}\right)+N_{D}-n\left(z\right)\right)$$

(13) $$\underset{v}{\sum }n_{v}\left(T\right)-N_{D}^{2D}\left[1-\frac{1}{1+\frac{1}{2}exp\left(\frac{E_{D}-E_{F}}{K_{B}T}\right)}\right]-\frac{\sigma_{s}}{e}\;=\;0$$

where $m^{*}$ and $\varepsilon_{r}$ are respectively the effective mass and the relative dielectric constant. $E_{c}$ is the total potential energy. The details of this model are well presented in the reference [31].

The interfacial density of total spontaneous and piezoelectric polarization charges at the InGaN/GaN can be written as:

(14) $$\sigma_{s}\;=\;P_{sp}\left(InGaN\right)+P_{pz}\left(InGaN\right)-P_{sp}\left(GaN\right)$$

For InGaN, the spontaneous and piezoelectric polarizations are expressed by [19]

(15) $$P_{sp}\left(InGaN\right)\;=\;0.003x+0.029\;C/m^{2}$$

(16) $$P_{pz}\left(InGaN\right)\;=\;-0.179x\;C/m^{2}$$

The spontaneous polarization for GaN is equal $P_{sp}\left(GaN\right)$ = −0.029 C/m^2^.

### 2.4. Numerical Method

The discretization of Schrodinger and Poisson equations has been performed using finite difference method. A centered second order scheme is used. Therefore, a continuous term such as $\frac{d}{dz}\left(f\frac{d\psi }{dz}\right)$ is discretized according to:

$$\frac{d}{dz}\left(f\frac{d\psi }{dz}\right)\;=\;\frac{\frac{f_{i+1}+f_{i}}{2}\frac{\psi_{i+1}-\psi_{i}}{\Delta z}-\frac{f_{i}+f_{i-1}}{2}\frac{\psi_{i}-\psi_{i-1}}{\Delta z}}{\Delta z}$$

The Schrodinger equation becomes: $H\psi_{i}\;=\;E\psi_{i}$. The non-zero elements of the matrix H are:

(17) $$H\left(i,j\right)\;=\;\left\{ \begin{array}{c} -\frac{\hbar^{2}}{2m_{0}\Delta z^{2}}\;\frac{1}{2}\left(\frac{1}{m^{*}\left(i\right)}+\frac{1}{m^{*}\left(i-1\right)}\right)\;if\;j\;=\;i-1\\ \frac{\hbar^{2}}{2m_{0}\Delta z^{2}}\left(\;\frac{1}{2}\left(\frac{1}{m^{*}\left(i\right)}+\frac{1}{m^{*}\left(i-1\right)}\right)+\frac{1}{2}\left(\frac{1}{m^{*}\left(i\right)}+\frac{1}{m^{*}\left(i+1\right)}\right)\right)+\;E_{c}\left(i\right)\;if\;j\;=\;i\\ -\frac{\hbar^{2}}{2m_{0}\Delta z^{2}}\frac{1}{2}\left(\frac{1}{m^{*}\left(i\right)}+\frac{1}{m^{*}\left(i+1\right)}\right)\;if\;j\;=\;i+1 \end{array}\right.$$

It is straightforward to obtain the matrix system related to the Poisson equation.

The above eigenvalues system and linear system are coupled and should be solved using an iterative method. The convergence is obtained when the difference on the Fermi level associated to two consecutive iterations is smaller than $10^{-4}\;\mathrm{eV}$. The numerical code has been validated in previous works [31].

### 2.5. Boundary Conditions

The boundary conditions related to Schrodinger equation are:

(18) $$\psi_{\nu ,k_{z}}\left(z\;=\;0\right)\;=\;\psi_{\nu ,k_{z}}\left(z\;=\;L\right)\;=\;0$$

where L is the total height of the structure.

The boundary conditions related to Poisson equation are:

(19) $${\frac{d(V_{H}+V_{P})}{dz}|}_{z\;=\;0}\;=\;{\frac{d(V_{H}+V_{P})}{dz}|}_{z\;=\;L}\;=\;0$$

## 3. Results and Discussion

The previous model is elaborated to investigate numerically the electronic and the electrical parameters of an In_x_Ga_1−x_N/GaN MQW. The electronic results deal mainly with:

- The longitudinal profile of the conduction band energy along the growth direction *z* for the two values of the number of QWs and several values of the temperature;
- The evolution of the conduction band offset Δ*Ec* versus the temperature for several values of the indium content;
- The variation of the Fermi energy and the 2DEG distribution at the interface versus the temperature, the indium content and the number of QWs;

In the matter of the electrical results, they concern mainly:

- The variation of the efficiency and short-circuit current density versus the number of QWs, the temperature, and In-content;
- The dependence of the open circuit voltage versus the temperature.

**Figure 2:**
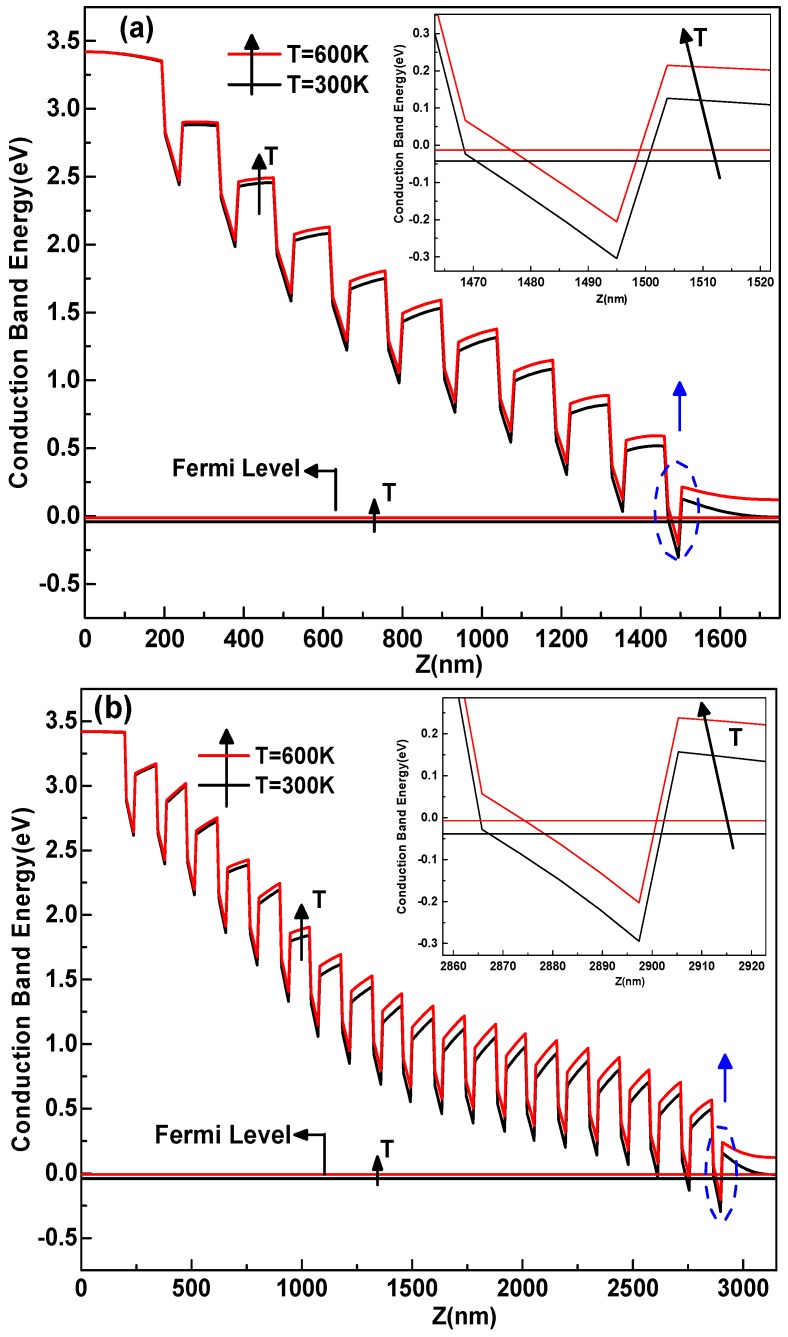
Conduction band energy along the growth direction z for the two heterostructures 10-periods (**a**), and 20-periods-In0.2Ga0.8N/GaN-MQW (**b**), with respect to temperature.

Figure 2a,b depicts the band diagram of the conduction band as a function of the growth direction z. In fact, the conduction band energy and the Fermi level were calculated by the self-consistent model, for two temperature values, of the 10 and 20-periods-In_0.2_Ga_0.8_N/GaN-MQW. A similar structure was made in [32]. Under this specific situation with polarization, the degree of the band diagram of tilting becomes higher and the quantum wells have a triangular shape of the situation without polarization [23,33]. 

We observed a variation of the conduction band by changing the temperature ranging from 300 to 600 K. This shift is caused by the reduction of the band gap at high temperatures. Furthermore, in our model, we also considered the variation of the effective mass and the dielectric constant of temperature which also influence the band structures Equations (12) and (15). Thus, we notice both the strong influence of the temperature of the energy of the conduction band and the Fermi energy. Hence, the device performance could be affected [34]. 

**Figure 3:**
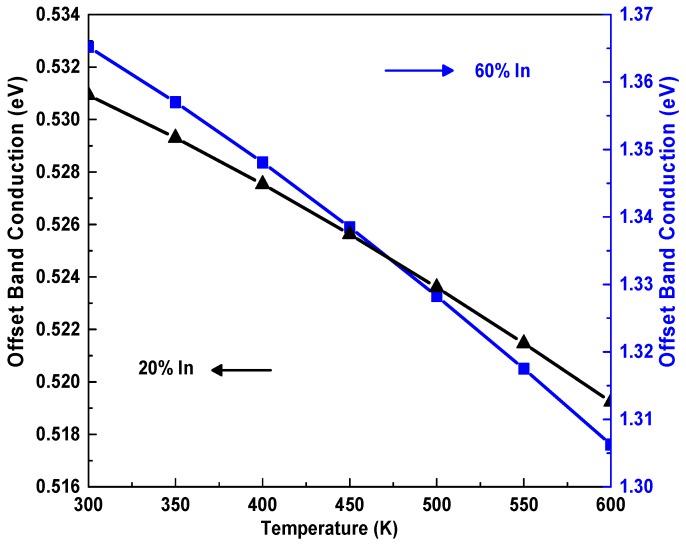
The conduction band offset Δ*Ec* dependence of temperature, and indium rate for In_x_Ga_1−x_N/GaN.

**Figure 4:**
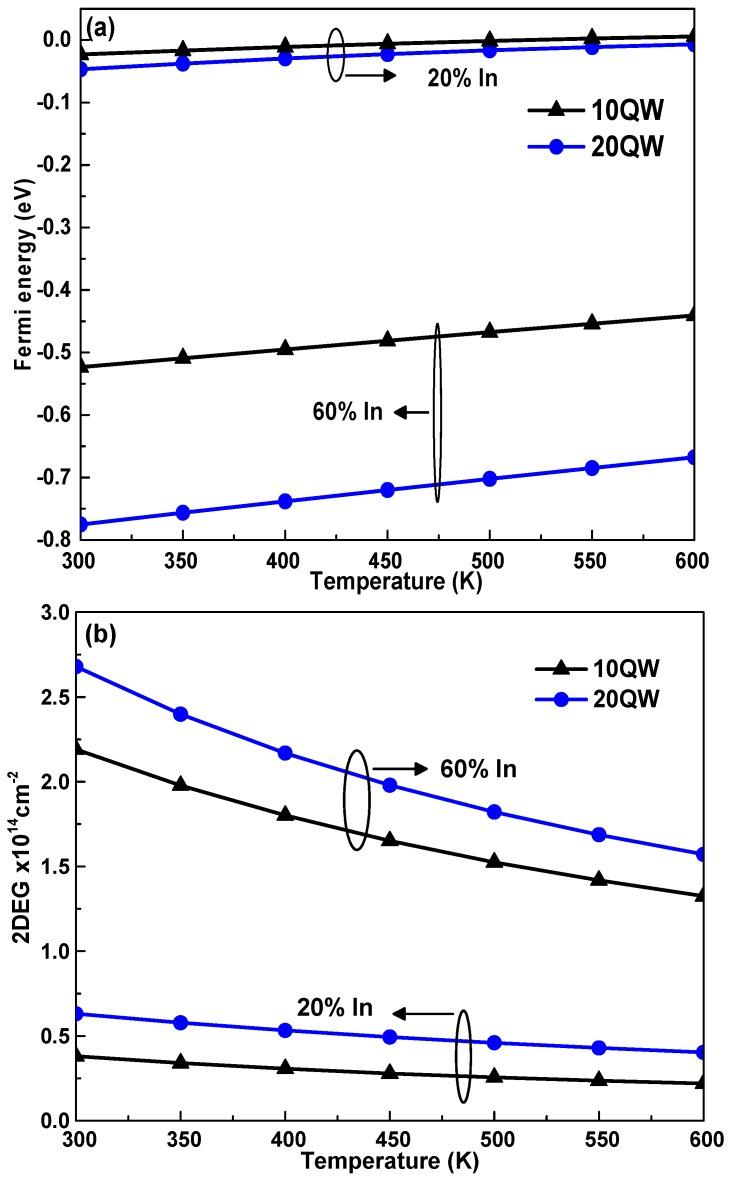
The dependence of the Fermi energy (**a**) and the 2DEG distribution at the interface (**b**) on temperature, indium rate and number of QWs.

Figure 3 shows the temperature dependence of conduction band offset Δ*Ec* for the In_x_Ga_1−x_N//GaN heterostructures for two indium fractions. The conduction band offset was evaluated as $\Delta E_{c}\;=\;0.7\left(E_{g}{}_{InGaN}\left(T,x\right)-E_{g}{}_{GaN}\left(T,x\right)\right)$. It is clear that increasing the temperature caused a degradation of the Δ*Ec* between the two InGaN and GaN materials. On the one hand, by increasing the indium rate from 20% to 60%, the Δ*Ec* increased from 0.532 to 1.358 eV at room temperature [35]. The increase in indium content improves carrier transport between the InGaN/GaN interfaces because the conduction band offset at the interfaces becomes larger [31]. We notice from Figure 4a that the Fermi energy increases within a temperature range from 300 to 600 K. On the other hand, the Fermi energy decreases due to the increase in the indium rate and the number of wells. The two processes which are responsible for the increase of Fermi energy as a function of temperature are the rise of the residual ionized donors and the electron traps. 

In InGaN/GaN heterostructures, the presence of piezoelectric and spontaneous polarization allows 2D electron gas density to be narrowed at the QWs region [23]. Additionally, In_x_Ga_1−x_N//GaN heterostructures have a larger conduction band offset, which collectively adds to the formation of high 2DEG density. The 2DEG sheet density in undoped In_x_Ga_1−x_N/GaN can be calculated by [31]. The change of the conduction band under the influence of temperature, indium fraction and the number of wells will cause a variation of the Fermi level and consequently a modification of 2DEG characteristics [36]. The temperature probably affects the 2D electron gas density confined in the quantum wells, Figure 4b. In fact, in the case of 60% indium fraction, 10-QW solar cell heterostructures, and within the temperatures ranging from 300 to 600 K, the 2DEG density decreased from 2.1910^14^ to 1.3210^14^ cm^−2^. The decrease in 2DEG density is caused by the narrowing of the conduction band offset at the InGaN/GaN interface as the temperature is increased [37]. On the contrary, for the value of indium rate x = 20% and 60%, the increase the number of wells tends to improve the two-dimensional electron gas density located at the hetero-interface. It should be noted that the increase in the number of periods leads to a rise of 2DEG density which is due to the enhancement of the carrier generation mechanism in the active region MQW, and the corresponding carrier confinement characteristics can also be improved [20]. These data indicate that the mechanism is strongly temperature-dependent. Consequently, this structure will be insensitive to variations in temperature, which eliminates the thermal drifts of devices based on InGaN/GaN heterostructures. Increasing In-rate and the QW number improves the recombination mechanism and electrical parameters of the solar cell. The 2DEG could also influence the Fermi energy. This behavior was investigated by Gazzah et al. [31] by a numerical model that combines the heat transfer equation and electronic properties of In_x_Ga_1−x_N/GaN heterostructures. They showed that the increase of the indium fraction improves the electronic properties and, consequently, an increase in heat dissipation. The novelty of this numerical model lies in taking account of the influence of Fermi level energy on the electrical parameters of the solar cell. However, according to Equation (2), these parameters also affect the electrical characteristics of the In_x_Ga_1−x_N-MQW solar cell.

The increase in temperature disrupts the characteristics of InGaN/GaN-MQW solar cells and their crystalline quality. Furthermore, when the temperature was increased, the Jsc increased slightly because of the reduction of the band gap and, consequently, a degradation of the open circuit voltage [9]. This phenomenon is due mainly to the limitation of the recombination mechanism at high temperature. This may result in a small collection of carriers due to reduced band offset at the interface of the well/barrier material. However, when the indium rate in the well material In_x_Ga_1−x_N is increased, the cell has to absorb more light and the absorption spectrum shifted towards longer wavelengths [19]. It is clear that the electronic properties are enhanced by increasing the indium fraction and the number of quantum wells for high-temperature conditions. These parameters also affect the electrical characteristics of the In_x_Ga_1−x_N-MQW solar cell. In order to validate our results at room temperature, we fixed the indium fraction x = 0.6, this choice was justified by Deng et al. [18]. Who found for a solar cell In_x_Ga_1−x_N /MQW, by varying the number of wells ranging from 10 to 50, that the maximum optimal efficiency is for a fraction of indium x = 0.6. However, these authors did not consider the impact of charge polarization on the electrical parameters of the cell [18]. One can say that taking account this effect in our model is beneficial for the performance of the cell.

**Figure 5:**
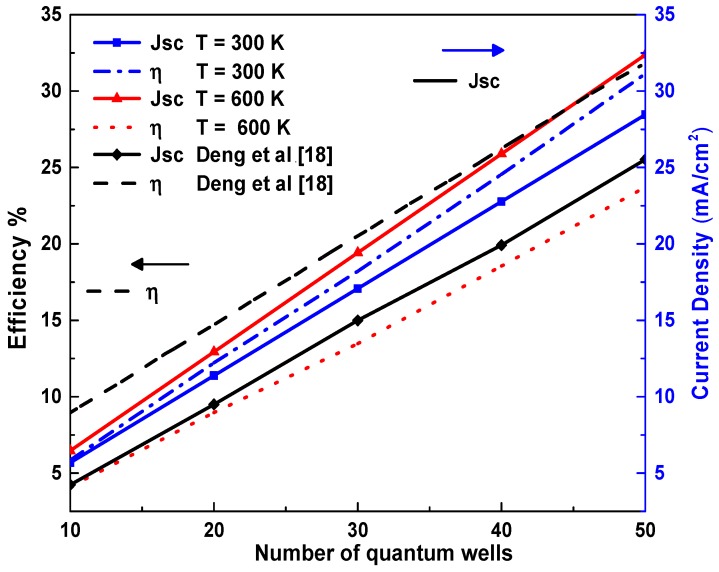
Dependence of η and Jsc on the QW number, at the temperatures 300 K, 600 K and the In-content is 60%.

Figure 5 depicts the evolution of the efficiency η and the current density Jsc as a function of the number of the wells and for T = 300 K and T = 600 K. On the one hand, the increase in Jsc is due mainly to the narrowing of the band gap resulting from the doubling the temperature. In this case, more carriers are generated in the region of QWs, and the flow of these carriers is reinforced by the thermal contribution [10]. On the other hand, the efficiency  η is degraded when raising the temperature. For an ambient temperature, our results are in good agreement with [18] for an In_0.6_Ga_0.4_N/GaN MQW-based solar cell with a 10 nm barrier thickness and a 4-nm well thickness. These authors also indicate that for this structure, the maximum efficiency is reached for an indium optimal fraction of 60% and for 50 QWs. In this situation, we found a better efficiency equal to 31%. In another numerical study conducted by Belghouthi et al. [19], the authors found an efficiency of 19% of 30 periods-In_0.6_Ga_0.4_N/GaN SC. The present results lead to an almost equal value of the efficiency. It is clear that the increase in the number of wells in high-In-content materials tends to improve the efficiency η and the Jsc [16,17]. In fact, increasing the number of wells and indium fraction for high temperatures tends to increase the confinement of the 2DEG gas density at quantum wells, which will improve the recombination process and therefore the cell’s performance. 

The degradation of the electronic properties at high temperature is mainly caused by the thermal generation of carriers in the InGaN-MQW region. These thermal photo-generated carriers will increase the leakage current, which is proportional to the saturation current $J_{QW0}$ [2,38]. In fact, the increase in temperature will decrease the probability of recombination of the photo-generated carriers; this effect will cause a limitation of the performance of the InGaN/GaN solar cell. To enhance the recombination of photogenerated carriers in MQW, and consequently, to improve the solar cell performance, increasing the indium fraction and the number of wells is a good solution [39].

**Figure 6:**
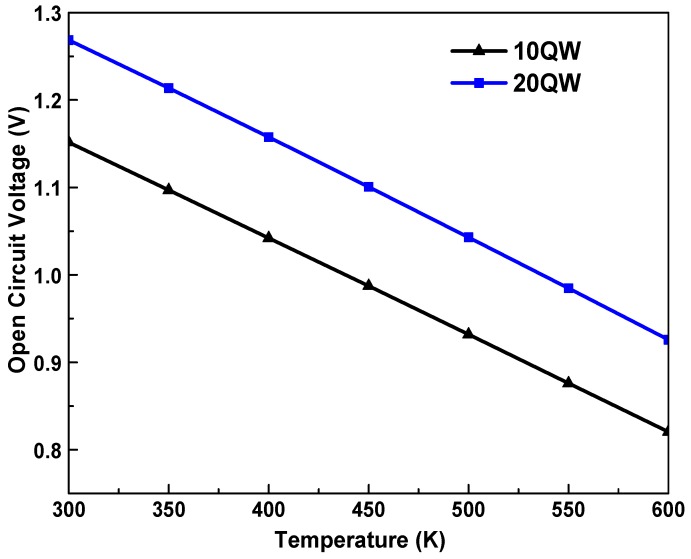
Dependence of the open circuit voltage Voc on the temperature for two values of the number of quantum wells (10 and 20 QWs).

**Figure 7:**
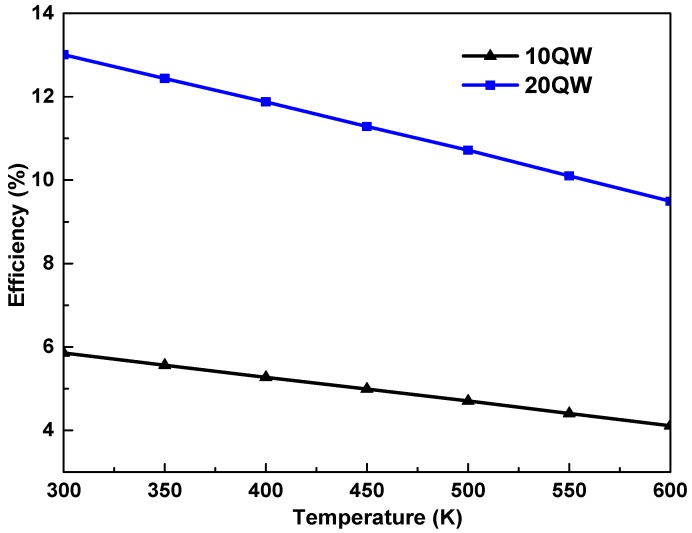
Efficiency dependence on the temperature for 10 QWs and 20 QWs.

In Figure 6 we plot the Voc as a function of the temperature of the solar cell In_0.6_Ga_0.4_N/GaN. The Voc is observed to deteriorate as the temperature is increased. The reduction of Voc is attributed to the limitation of the recombination mechanism in high temperatures [9,10]. It worth mentioning that increasing the QWs number from 10 to 20 QW tends to increase the Voc from 1.15 to 1.26 V. At higher temperatures, the short circuit current increases slightly, whereas the open circuit voltage drops significantly. However, the solar cell efficiency is seriously degraded due to a large voltage drop at high temperature. The Voc and the current density Jsc are the main parameters for determining the efficiency. The dependence of the η on the QWs number and temperature is apparent in Figure 7 which demonstrates that raising the temperature causes a degradation in the efficiency of the cell. In fact, for the high fraction of indium 60%, by increasing the number of wells will probably reduce the negative effect of the temperature which limits the electronic properties. Hence, the 2DEG gas density improves and consequently, the performance of the cell improves too. 

## 4. Conclusions

In this work, a numerical model has been developed based on the 1D self-consistent Schrödinger–Poisson equations coupled with the electrical parameters of InGaN-MQWSC. The numerical treatment based on the finite difference method takes into consideration the effect of spontaneous and piezoelectric polarization. The simulation results reveal the ultimate effect of temperature, In-content, and the QW number. These factors may result in either improve or deteriorate the overall device performance and its electronic proprieties. It has been proven that the increase of the temperature for a range from 300 to 600 K caused a decrease in the parameters of the cell (the Voc and the efficiency) due to the degradation of the electronic properties such as the energy of the Fermi level, the band gap, the 2DEG density, and the offset conduction band. Furthermore, an InGaN/GaN MQWSC of higher In-rate, and increased quantum well numbers improves the electronic proprieties and efficiency of the solar cell for operations at a higher temperature. The results give the opportunity to manufacture high efficiency nitride-based solar cells. In addition, this model could be coupled to thermal and optical properties of InGaN/GaN solar cells at the nanoscale in 2D and 3D model simulation in future work.

We intend in the future to investigate heat generation and nano heat transfer using a Dual Phase Lag (DPL) model. The effect of the cooling of solar cells will also be investigated.
